# Emerging innovations in electrically powered artificial muscle fibers

**DOI:** 10.1093/nsr/nwae232

**Published:** 2024-07-05

**Authors:** Tianhong Lang, Lixue Yang, Shiju Yang, Nan Sheng, Yiyao Zhang, Xiaofei Song, Yang Guo, Shaoli Fang, Jiuke Mu, Ray H Baughman

**Affiliations:** Key Laboratory of Mechanism Theory and Equipment Design of Ministry of Education, School of Mechanical Engineering, Tianjin University, Tianjin 300350, China; Key Laboratory of Mechanism Theory and Equipment Design of Ministry of Education, School of Mechanical Engineering, Tianjin University, Tianjin 300350, China; Key Laboratory of Mechanism Theory and Equipment Design of Ministry of Education, School of Mechanical Engineering, Tianjin University, Tianjin 300350, China; Key Laboratory of Mechanism Theory and Equipment Design of Ministry of Education, School of Mechanical Engineering, Tianjin University, Tianjin 300350, China; Key Laboratory of Mechanism Theory and Equipment Design of Ministry of Education, School of Mechanical Engineering, Tianjin University, Tianjin 300350, China; Key Laboratory of Mechanism Theory and Equipment Design of Ministry of Education, School of Mechanical Engineering, Tianjin University, Tianjin 300350, China; College of Materials Science and Engineering, Donghua University, Shanghai 201620, China; Alan G. MacDiarmid NanoTech Institute, University of Texas at Dallas, Richardson, TX 75080, USA; Key Laboratory of Mechanism Theory and Equipment Design of Ministry of Education, School of Mechanical Engineering, Tianjin University, Tianjin 300350, China; Alan G. MacDiarmid NanoTech Institute, University of Texas at Dallas, Richardson, TX 75080, USA

**Keywords:** fibers, artificial muscles, electrically powered actuation, electrochemical, smart textile

## Abstract

This review systematically explores the inherent structural advantages of fiber over conventional film or bulk forms for artificial muscles, emphasizing their enhanced mechanical properties and actuation, scalability, and design flexibility. Distinctive merits of electrically powered artificial muscle fiber actuation mechanisms, including electrothermal, electrochemical and dielectric actuation, are highlighted, particularly for their operational efficiency, precise control capabilities, miniaturizability and seamless integration with electronic components. A comprehensive overview of significant research driving performance enhancements in artificial muscle fibers through materials and structural innovations is provided, alongside a discussion of the diverse design methodologies that have emerged in this field. A detailed comparative assessment evaluates the performance metrics, advantages and manufacturing complexities of each actuation mechanism, underscoring their suitability for various applications. Concluding with a strategic outlook, the review identifies key challenges and proposes targeted research directions to advance and refine artificial muscle fiber technologies.

## Teaser

This review paper outlines the evolution of electrically powered artificial muscle fibers, highlighting recent advancements in base materials, driving mechanisms, and structural design, and offers insights into future development trends.

## INTRODUCTION

The pursuit of biomimetic actuation systems has propelled substantial progress in materials science and engineering, culminating in the innovative development of artificial muscles [1–3]. These synthetic constructs are engineered to mimic the contractile function of biological muscles, heralding a transformative era in robotics [4,5], wearable technologies [6,7] and biomedical devices [8]. The evolution of artificial muscles has shifted from basic mechanical designs to advanced materials capable of dynamic, responsive actuation. Artificial muscle fibers, in particular, represent the pinnacle of this developmental arc, providing an ideal combination of mechanical properties and actuation functional adaptability. Their extensible form not only allows for exceptional mechanical flexibility but also supports precise emulation of natural muscle movements, making them ideal for applications that require complex manipulation and adaptive functionality [9,10].

Among various actuation mechanisms, electrically powered artificial muscles, which utilize electrical energy to generate mechanical motion, mark a significant improvement over absorption, light-powered artificial muscle and conventional pneumatic or hydraulic systems [11–14]. These muscles offer fine control, rapid response times and greater compactness, facilitating easier integration across a spectrum of applications from delicate medical instruments to robust robotic assemblies [8]. What makes them particularly appealing is their ability to scale and integrate with electronic controls, leading to smarter, more autonomous devices [15,16].

This review focuses on three principal types of electrically powered artificial muscle fibers (EAMFs): electrothermal, electrochemical and dielectric actuation mechanisms. Each technology utilizes electricity to induce movement differently, ranging from thermal expansion and contraction in electrothermal muscles, to ion migration in electrochemical muscles, to electric-field-induced deformation in dielectric elastomers. Specifically, we will detail the mechanisms of three types of EAMFs and explore the strengths and weaknesses of their performance characteristics at the mechanistic level. Additionally, we will comprehensively map the milestone developments in the areas of material expansion, multi-scale structural design, device development and potential applications for these artificial muscle fibers. Finally, considering the significant challenges confronting the advancement of this field, our understanding prompts us to propose some future perspectives for the development of different types of electrically driven artificial muscle fibers, hoping to inspire further research in this area.

## WHY FIBER?

**Figure 1.**
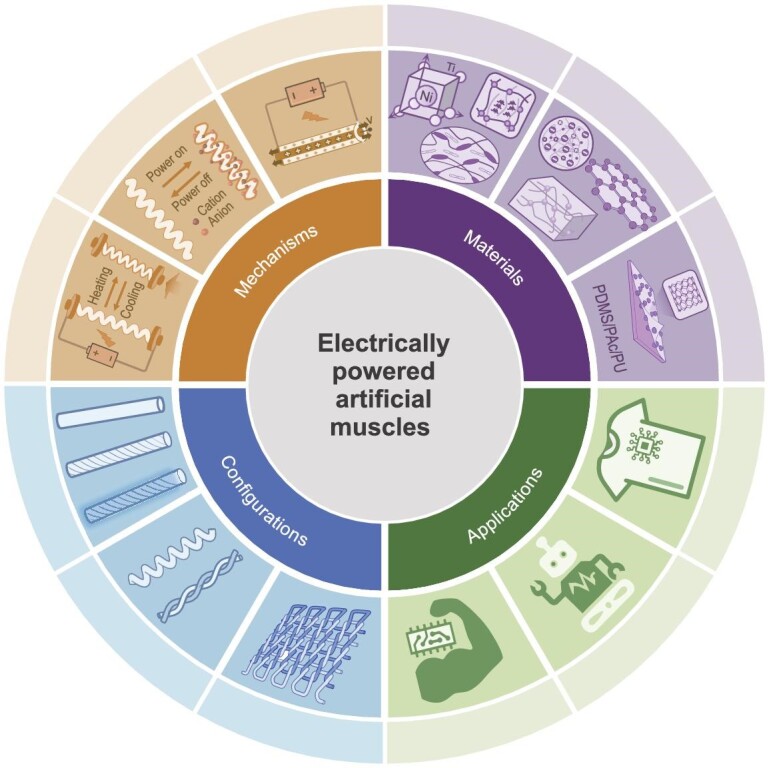
Schematic of the EAMFs categorized from the mechanism, material components and configurations, as well as their application fields.

The field of artificial muscle development has been revolutionized by the development of artificial muscle fibers, which represent a significant advancement toward replicating the functionality of biological muscles with advanced structural sophistication and actuation performance [1–4]. This section explores the intrinsic advantages of artificial muscle fibers, focusing on their exceptional structural adaptability [6,7], superior mechanical performance [8] and high biomimetic fidelity [5]. These attributes demonstrate the transformative potential of these systems in reshaping actuation technology (Fig. 1).

**Figure 2.**
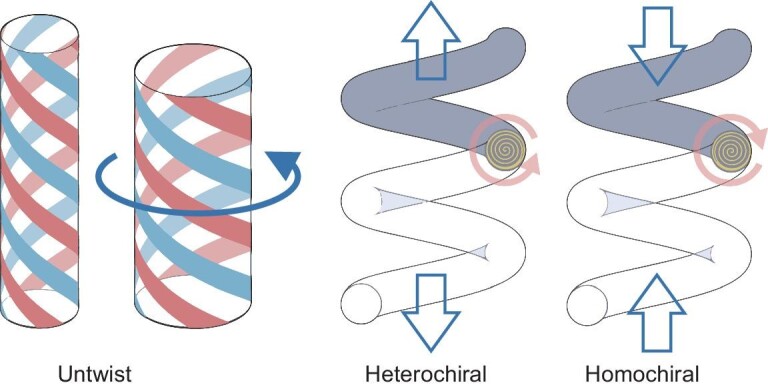
Schematic illustration of the mechanism by which torsional fiber actuation drives tensile actuation for heterochiral and homochiral coiled fibers. Reproduced from refs. [49] (Copyright 2014, AAAS) and [75] (Copyright 2014, AAAS) with permission.

Although different stimulation mechanisms result in varied volume changes of active materials, fiber-formed artificial muscles share similar deformation rules. For instance, we can take polymer artificial muscle fibers as an example. Initially, the polymer chains within the fibers are aligned parallel in the fiber direction. Upon twisting, these chains reorient into a helical configuration, establishing a bias angle relative to the fiber's original orientation (Fig. 2). When subjected to heating or other stimulation that provides expansion in polymer diameter, the polymer chains in the twisted fiber untwist. The extent of untwist upon stimulation can be quantified by the change in turns per unit length of the fiber (ΔT). This untwisting is crucial as it generates torsional actuation; the fiber produces a mechanical torque that modifies its physical configuration. For torsionally tethered coiled fibers, this torsional fiber untwist alters the coil helix angle by transferring fiber twist to the twist of coiling, leading to a substantial change in the length of spring (ΔL, Equation (1)), either contracting or expanding depending on the relative chirality of fiber twist and coiling. For homochiral fibers, where the chirality is the same for fiber twist and coiling twist, the untwisting torque draws the coils closer, resulting in length contraction. Conversely, for heterochiral coils with opposite twist and coiling chirality, the transfer or fiber twist to the twist of coiling during heating extends the coil length, since this fiber untwist cancels some of the twist of coiling.


(1) \begin{eqnarray*}
\Delta T = \frac{{N\Delta L}}{{{{l}^2}}}
\end{eqnarray*}

For a coil of *N* turns and length *L* made from a fiber of length *l*, assuming negligible change in fiber length, the relationship between changes in fiber twist and changes in coil length can be described by Equation (1).

During the production process, fibers inherently promote molecular alignment along their length, which optimizes mechanical properties in the direction of tension [9,17]. This alignment increases tensile strength and flexibility, advantages that are difficult to replicate in films or bulk materials with more isotropic molecular orientations. Additionally, the ability to control the microstructure of fibers through methods such as stretching, twisting or weaving allows for the customization of mechanical properties by modifying fiber density or microstructural configurations, enhancing overall strength and toughness [10–12].

Moreover, artificial muscle fibers are inherently scalable, produced at scales from micro to macro without losing functional integrity or actuation performance [11,18]. This scalability, along with fibers’ adaptability, allows for their integration into a variety of systems and materials such as smart textiles and soft robotics [10,12]. Advanced manufacturing techniques, including electrospinning, coaxial spinning, wet spinning, thermal drawing and 3D printing, further refine fiber precision and reliability while integrating advanced materials like conductive polymers (CPs), carbon nanotubes (CNTs) and graphene [19–23].

In conclusion, artificial muscle fibers leverage the unique advantages of fiber morphology—structural adaptability, enhanced mechanical properties and biomimetic functionality—to provide superior performance over films or bulk materials. Their flexibility, efficiency and innovative manufacturing capabilities underscore their potential to transform a broad spectrum of technological applications, making them an optimal choice for the next generation of actuation technology.

## ACTUATION MECHANISMS OF ELECTRICALLY POWERED ARTIFICIAL MUSCLE FIBERS

EAMFs represent a significant leap forward in muscle technology, characterized by high motion precision, adaptable size, straightforward cluster design and efficient energy conversion (qualities that are particularly pronounced in electrochemical-powered artificial muscles). This article delves into the three primary mechanisms of EAMFs: electrothermal, electrochemical and dielectric. Each mechanism is defined by unique operating principles and specific strengths and weaknesses that cater to different application needs: electrothermal mechanisms are valued for their rapid response and high power density; electrochemical mechanisms are noted for precise control and superior energy efficiency; and dielectric mechanisms excel in high-frequency operation. Together, these technologies establish a robust scientific base and wide-ranging application potential for the continued development of EAMFs, positioning them as a versatile solution in the evolution of artificial muscle technology.

### Electrothermal actuation

Electrothermal actuation stands as a cornerstone in the development of artificial muscle fibers, expertly converting electrical energy into controlled thermal expansions and contractions to mimic the dynamic functionalities of natural musculature [24,25]. This section explores the foundational principles, groundbreaking material innovations, and structural advancements that characterize this transformative technology, illustrating the strides made through multidisciplinary research in the electrothermal muscle domain.

The mechanism of electrothermal actuation involves the precise application of electrical current to artificial muscle fibers. This current, passing through or alongside electrothermal conductive materials, induces Joule heating. The resultant temperature increase causes thermally active materials within the fibers to expand. Conversely, reducing or ceasing the electrical stimulus allows the fibers to cool and contract, returning them to their original state. A crucial feature of this mechanism is the intrinsic twisted configuration of the fibers, which not only elongates but also untwists upon thermal expansion, thus amplifying the actuation effect through a combination of axial stretching and rotational movements as they expand volumetrically.

Electrothermal artificial muscle technology is categorized into two primary types based on the source of thermal actuation. Direct electrothermal muscles comprise fibers that directly convert electrical energy into thermal energy, facilitating actuation intrinsically. Conversely, Hybrid electrothermal muscles represent a composite approach, where thermally active materials are intricately integrated with electrothermal conductive materials. This synergistic set-up leverages the conductive properties of the electrothermal fibers to distribute heat evenly throughout the polymer, ensuring uniform thermal expansion and effective actuation.

Material selection and innovations: the domain of electrothermal artificial muscle fiber development showcases a diverse array of material innovations, from nickel-titanium (NiTi) alloys to advanced polymer-based systems. This evolution reflects a strategic shift towards materials that offer both high thermal responsiveness and electrical conductivity, aiming to replicate the dynamic functions of biological musculature with increased precision and efficiency.

#### NiTi alloy fibers

**Figure 3.**
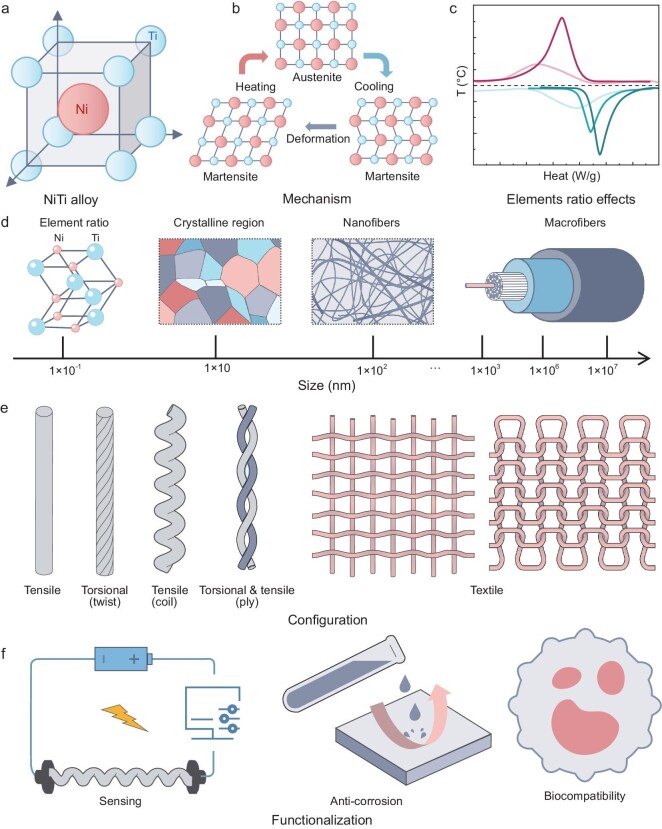
The composition, actuation mechanisms and historical development of shape memory alloy (SMA) artificial muscle fibers. (a) Composition and (b) actuation mechanisms. (c) Fine-tuning the activation temperatures. (d) Multi-scale fabrication. (e) Actuation modes expansion. (f) Enhancing durability.

The inception of electrothermal artificial muscles can be traced back to the use of NiTi alloys, renowned for their shape memory properties. The unique actuation mechanism of NiTi shape memory alloy (SMA) fibers is primarily attributed to their reversible phase transformation between martensitic (low temperature) and austenitic (high temperature) states, a process that is thermally induced through Joule heating (Fig. 3a and b). This transformation enables the fibers to contract and expand in a manner reminiscent of biological muscle action, providing a robust model for artificial muscle design.

*Customized activation temperatures.* Fine-tuning the activation temperatures of NiTi SMA fibers has emerged as a crucial development, enabling the customization of their response for tailored applications. Researchers have achieved this by making precise adjustments in the alloy's composition and optimizing thermal treatment processes as shown in Fig. 3c [26,27]. This meticulous approach has allowed for the manipulation of the temperature range within which NiTi SMA fibers undergo their characteristic martensitic-austenitic phase transformation [28]. Achieving such a level of control over activation temperatures opens up new possibilities for integrating NiTi SMA fibers into systems that demand exact thermal activation thresholds.

*Multi-scale fabrication.* The field of NiTi SMA fiber has seen remarkable advancements with the development of fibers across various diameters, enabling their application in a multitude of domains (Fig. 3d). Recent advancements in microscale fabrication have propelled NiTi SMA fibers into the forefront of micro-actuator and micro-robotics research [29,30]. Techniques such as focused ion beam (FIB) milling [31] have enabled the production of NiTi SMA fibers with precise dimensional control, significantly enhancing their actuation responsiveness and compatibility with microelectronic and microfluidic systems.

*Expanding actuation modes.* The development of NiTi SMA fibers has revolutionized the capabilities of artificial muscles, extending their actuation modes beyond simple contraction to include bending, and notably, rotational movements through twisting and coiling (Fig. 3e) [32–34]. For instance, torsional actuators derived from twisted NiTi SMA fibers have found applications in soft robotics, where they enable the creation of more lifelike and adaptable robotic limbs and joints [35,36]. Following the expansion of actuation modes, recent innovations have further explored the potential of weaving NiTi SMA fibers to achieve 3D, anisotropic deformations [30,37]. This technique allows for the construction of artificial muscles that can perform complex movements and adapt to varying loads, emulating the multifunctionality of natural muscle tissue.

Integrating SMA fibers with textile and weaving techniques can enhance their movement capabilities and expand their application spectrum, particularly in smart textiles and soft robotics [38]. A notable innovation, the ‘constrained weaving’ technique, involves weaving SMA fibers into a matrix with strategically placed apertures, allowing for substrate-free actuation and enabling precise, periodic directional movements [39]. Specifically, this method utilizes the controlled heating of SMA fibers to achieve deterministic curvilinear actuations. In another wearable technology, embedding SMA fibers into fabrics with a negative Poisson's ratio has enhanced adaptability in size and shape while providing variable haptic feedback through electrical current adjustments [40]. Similarly, further advancement is the development of knot-architectured fiber actuators (KAFA), which feature a knotted structure for robust mechanical integrity and self-locking capabilities [41]. Activated by Joule heating, these actuators can exert significant forces and achieve high strains, demonstrating the potential of SMA braided actuation technology to advance the field of smart textiles and contribute innovative approaches to the design and evolution of intelligent systems.

*Enhancing durability.* The susceptibility of NiTi SMA fibers to oxidation at elevated temperatures poses a challenge to their longevity and performance. To address this, recent research has focused on developing oxidation-resistant coatings that safeguard the fibers without impeding their actuation capabilities (Fig. 3f). Innovations in coating materials, including ceramic and nanocomposite layers, have shown promise in extending the operational lifespan of NiTi SMA fibers, ensuring consistent performance across a wide range of temperatures and environmental conditions [42,43].

#### Polymer fibers

**Figure 4.**
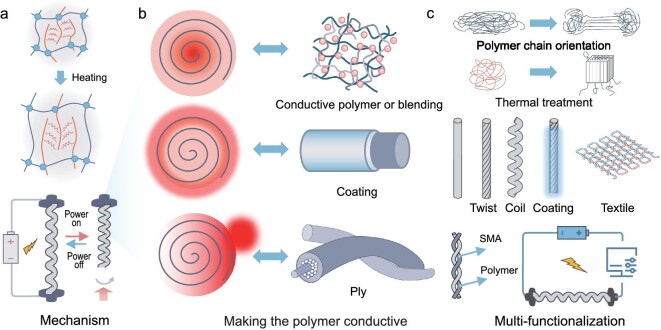
(a, b) The actuation mechanisms of polymer-based artificial muscle fibers. (c) Enhancement strategy and functionalization.

Polymer fibers have marked a significant evolution in the field of artificial muscles, evolving from reliance on metal alloys to the adoption of more adaptable and versatile polymeric materials. Initially, actuation within polymer-based systems was predominantly thermal, capitalizing on the inherent property of polymers to expand upon heating (Fig. 4a) [25,44]. While effective, this method encountered limitations in terms of control precision, responsiveness and the need for external heat sources, which could be impractical for many applications. The introduction of electrothermal actuation techniques represented a paradigm shift in the design and operation of polymer-based artificial muscles (Fig. 4b). The integration of conductive materials with polymers through blending conductive materials, applying conductive coatings, or intertwining with conductive fibers or sheets, has revolutionized polymer-based artificial muscle fibers by enabling precise electrothermal actuation. For composite blend materials, conductive materials form an electrical network within a matrix that possesses thermal expansion properties. When a voltage is applied across this matrix, the conductive materials rapidly generate Joule heat and uniformly transfer this heat to the matrix material. This process initiates thermal expansion, which is then converted into actuation behavior in artificial muscle fibers [45,46]. For the coating case, this external layer acts as a resistive heater, where the electrical current flows through the coating, generating Joule heat [47,48]. The heat is then transferred to the underlying polymer, causing it to expand and actuate. This technique allows for the addition of electrical conductivity to non-conductive polymers without significantly altering their intrinsic properties. In particular, it also enables the use of high-performance polymers that may not be inherently conductive but possess desirable mechanical properties for artificial muscles. Similar to the coating case, intertwining conductive fibers or wrapping polymer fibers with conductive fiber or sheets creates targeted heating pathways, enabling precise electrothermal actuation through the rapid heating of these conductive elements upon electrical stimulation [49,50]. Furthermore, adjusting the topological structure of thermal active materials and heating conductive materials can further enhance the actuation performance of electrothermal artificial muscle fibers (Fig. 4c). Sheath-core configuration: our team has developed electrothermally driven sheath-run artificial muscles [51], where a thermally active polymer is coated onto conductive CNT fibers with a specific thickness ratio and then further twisted to produce a series of torsional and tensile artificial muscle fibers. This structure exhibits superior actuation performance compared to previous hybrid artificial muscles. The sheath-core configuration effectively absorbs the heat from the CNT core while facilitating rapid thermal exchange with the environment and efficiently utilizing the twisted potential energy of the CNT fiber surface layer. Consequently, this leads to improvements in actuation stroke, response speed and other actuation parameters.

In addition to enhancing the actuation performance of artificial muscle fibers, adding additional functionalities to these fibers has become a hot research topic in recent years.

An exemplary application of this concept is seen in the development of elastic fiber artificial muscles that utilize twisted natural rubber fibers coated with bent CNT sheets. These fibers are capable of multidirectional actuation and sensing from a single electrical signal [52]. Building on this, a sophisticated multilayered coaxial artificial muscle fiber has been developed, featuring a CNT fiber core encased within layers of elastomer, nanofiber networks and a Ti_3_C_2_T_x_ (Mxene, T_x_ represents surface termination)/CNT thin sheath [53]. This composite structure not only facilitates stretch and torsion actuation but also integrates sensing capabilities, mirroring the complex functionality of biological structures like snail tentacles. These innovations underscore the transformative potential of sheath-core and multilayered coaxial structures in advancing artificial muscles, enhancing actuation profiles and integrating sensory feedback, thus marking a significant advancement toward bio-inspired actuators.

Additionally, to address operational challenges in low temperatures (below 0°C), a novel coaxial artificial muscle fiber was created using electrospinning techniques to form polycaprolactone (PCL) nanofiber sheaths around CNT fiber cores [54]. The choice of PCL, with its low glass transition temperature, ensures that the muscle fiber maintains excellent performance in sub-zero conditions, offering a practical solution for applications that require functionality in extremely low-temperature environments. This example highlights that by carefully selecting and integrating these conductive elements, we can tailor the actuation properties of polymer-based artificial muscle fibers to meet specific requirements.

*Intertwining conductive fibers.* Combining the rapid response and electrical conductivity of SMA with the excellent drive stroke-to-temperature linearity of polymers can further enrich the motion patterns and drive control capabilities of artificial muscles. For instance, an innovative actuator has been developed by merging the properties of SMA fiber and nylon fiber. This hybrid design leverages the rapid actuation response and electrical conductivity of SMAs along with the high tensile strength and lightweight properties of polymer fiber. This combination effectively brings together the robust, quick-response characteristics of SMAs with the flexibility and resilience of polymer fiber, creating a versatile and powerful artificial muscle [34]. Similarly, by twisting multiple types of polymer fibers together, it is possible to cohesively blend the unique actuation properties of various fibers—such as elasticity, conductivity and thermal responsiveness—into a single composite fiber, thereby optimizing overall functionality. For instance, the development of an artificial muscle featuring a ‘rigid-flexible’ structure was achieved by co-twisting rigid heterocyclic aramid (HA) fibers with flexible silver-plated nylon fibers [55]. This HA@SPN coiled fiber muscle not only supports high loads and responds swiftly but also functions effectively at lower average temperatures, significantly improving its working and power densities.

*Molecular-level design.* In addition to the composite approach of integrating conductive and thermally responsive materials, the molecular-level design of polymers, specifically their orientation and crystallinity, plays a pivotal role in enhancing the performance of polymer-based artificial muscle fibers. For instance, polylactic acid (PLA) fibers, widely utilized in biomedical applications due to their biocompatibility and biodegradability, typically exhibit low anisotropy due to poor crystallinity, which limits their actuation capabilities. This issue was addressed by applying a tensile-oriented and annealed process to PLA fibers [56], significantly enhancing the anisotropy and thermal stability of the fiber's crystalline structure. This process facilitated the creation of high-performance poly(L-lactic acid)/poly(D-lactic acid) composite fibers, which, when co-twisted with metal wires, produce efficient artificial muscles. Similarly, poly(p-phenylene-benzimidazole-terephthalamide) fibers [57], recognized for their impact resistance, were optimized by incorporating single-walled carbon nanotubes (SWCNTs) as orientation seeds. This technique effectively induced a favorable alignment in the polymer fibers, resulting in artificial muscles with superior contraction rates when co-wound with CNT tapes. Such advancements demonstrate the potential for molecular engineering to significantly improve the functionality and performance of artificial muscle technologies.

**Figure 5.**
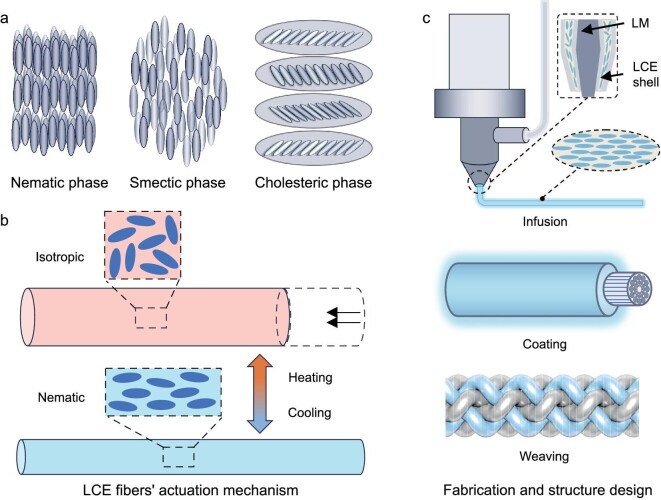
Response mechanisms of LCE-based electrothermal artificial muscle fibers, fabrication processes, structural forms and design concepts for conductive LCE muscle fibers. (a) Molecular architecture. (b) Working mechanism. (c) Fabrication and structure design. Reproduced from ref. [62] with permission. Copyright 2023 Wiley-VCH.

*Liquid crystal elastomers (LCEs).* LCEs represent an innovative class of polymers that blend the mechanical flexibility of elastomers with the optical and thermal responsiveness of liquid crystals, positioning them at the forefront of artificial muscle technology. LCEs are composed of a polymer network incorporating liquid crystal (LC) units, which can be attached to the polymer backbone or as side groups. These LC units can exhibit various phases, such as the nematic phase, where molecules are directionally aligned without regular arrangement, or the smectic phase, where molecules are organized into layers (Fig. 5a).

Heating an LCE typically induces a transition from an ordered nematic phase to a disordered isotropic phase. This transition leads to a contraction of the macroscopic shape of the LCEs fiber (Fig. 5b) [58]. Upon cooling, the material reverts to its original nematic phase, restoring its initial shape. Advancements in LCE fiber technology have seen significant developments, such as the successful fabrication of LCE fibers up to meter scale in length using direct ink writing (DIW) 3D printing technology [59]. In a quest for even finer scales, innovative scalable fabrication methods inspired by natural spider spinning techniques have been utilized to produce micron-sized LCE fibers, achieving diameters as small as 2.6 μm [60]. These microfibers are capable of rapid contraction and generate significant actuation forces under near-infrared light irradiation.

In the realm of electrothermal actuation, the incorporation of liquid metal (LM) as a filler has notably enhanced the responsiveness of LCE fibers, leading to fibers that exhibit remarkable contraction properties and fast response rates under electrical stimulation (Fig. 5c) [61]. Additionally, a self-recovering coiled artificial muscle fiber was developed by coating LCE layers on elastic CNT fibers, which demonstrated substantial contraction stroke capabilities and stability over thousands of cycles [15]. Further, the integration of LCE fibers with silver-plated nylon filaments through a braiding technique has produced a novel braided actuator, suitable for applications in artificial arms and soft pumps [62], showcasing its promising potential in smart wearables and soft robotics.

### Electrochemical actuation

The core mechanism of electrochemical actuation involves the movement of ions within and around a material when an electric field is applied. This ionic movement (ions de-/intercalation) results in a change in the volume of the actuator, leading to its expansion or contraction. This process is associated with changes in the electrolyte surrounding the actuator and, in specific categories (electro-responsive hydrogel), with the movement of solvent molecules. Here, we delve into the operational principles of electrochemical actuation, involving double-layer capacitance and pseudocapacitance such as redox reactions and ion de-/intercalation, and illustrate these concepts with examples of specific materials.

#### Pseudocapacitance in conductive polymers

**Figure 6.**
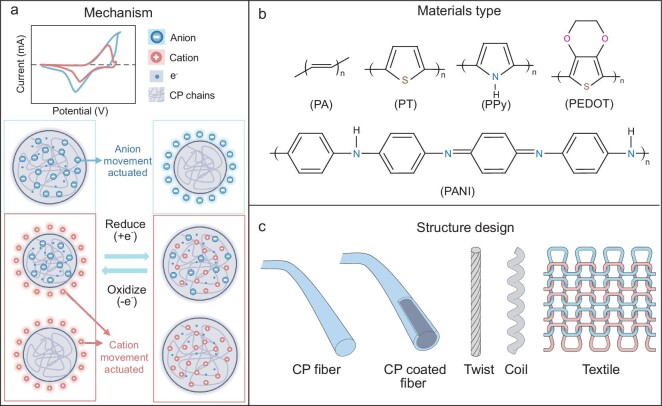
Response mechanisms of artificial muscle fibers based on pseudocapacitive principle, representative materials, fiber structures and design strategies. (a) Response mechanisms. (b) Representative materials. (c) Fiber structures and design strategies.

CPs can be crafted into fibers or yarns, forming artificial muscle fiber. The mechanism of action in these devices hinges on redox reactions, where the application of electric potential leads to electron gain (reduction) or loss (oxidation). This electron transfer is coupled with the movement of counter-ions into or out of the polymer, causing the material to swell or shrink and thereby actuate (Fig. 6a and b).

Specifically, most CP-electrochemical artificial muscles can be categorized into two types, p-doped and n-doped, based on the charge effects generated during the doping process. The actuation mechanism of CP artificial muscles can be summarized by three basic equations (Equations (2–4)).


(2) \begin{eqnarray*}
\mathop {(C{{P}^0}) + n\big(\alpha _{\textit{solv}}^ - \big)}\limits_{{\mathrm{Neutral}}\,{\mathrm{chains}}} \leftrightarrow \mathop {\left[(C{{P}^{n + }}){{{\big(\alpha _{\textit{solv}}^ - \big)}}_n} \right]}\limits_{{\mathrm{Oxidized}}\,{\mathrm{chains}}} +\, n{{e}^ - }
\end{eqnarray*}

Equation (2) and illustration 1 of Fig. 6a depict the mechanism of anion-driven p-doped CPs. During oxidation, electrons (e^−^) are drawn from the polymer chains, which generates positive charges. To maintain the charge balance, the small, mobile solvated anions (a^−^) permeate the CPs along with the solvent, leading to expansion. Conversely, when the polymer chains are reduced by the electron's injection, this causes the polymer to shrink as lots of mobile anions are moved out.


(3) \begin{eqnarray*}
&&\mathop {\left[(C{{P}^0}){{{({{A}^ - })}}_n}{{{(c_{\textit{solv}}^ + )}}_n}\right]}\limits_{{\mathrm{Neutral}}\,{\mathrm{chains}}} \leftrightarrow \mathop {(C{{P}^{n + }}){{{({{A}^ - })}}_n}}\limits_{{\mathrm{Oxidized}}\,{\mathrm{chains}}}\\
&&\qquad +\, n\big(c_{\textit{solv}}^ +\big) + n{{e}^ - }
\end{eqnarray*}

Equation (3) and Illustration 2 of Fig. 6a demonstrate the mechanism of cation-driven p-doped CPs, where large anions are embedded within the polymer chains. Since the large anions (A^−^) are trapped in the polymer, the smaller cations (c^+^) must act as charge-balancing mobile ions during the oxidation/reduction instead of anion de-/doping as well as solvent exchange for osmotic balance. These actuations can be summarized as the doping of cations occurring during reduction to swell the CP and the cation de-doping during oxidation to yield contraction.


(4) \begin{eqnarray*}
\mathop {(C{{P}^0}) + n\big(c_{\textit{solv}}^ + \big)}\limits_{{\mathrm{Neutral}}\,{\mathrm{chains}}} +\, n{{e}^ - } \leftrightarrow \mathop {\left[(C{{P}^{n - }}){{{\big(c_{\textit{solv}}^ + \big)}}_n}\right]}\limits_{{\mathrm{Reduced}}\,{\mathrm{chains}}}
\end{eqnarray*}

The mechanism depicted in Equation (4) and illustration 3 of Fig. 6a is demonstrated by n-doped CPs (such as thiophene and thiophene derivatives) and mobile cations (c^+^). For n-doping, electrons are injected into the polymer chains, generating negative charges. To maintain the charge balance, the small, mobile cations (c^+^) enter the CPs with the solvent to achieve osmotic balance. Thus, CPs swell during the cation-permeated reduction and contract during oxidation.

*Materials and development progress.* Spinning, particularly wet spinning, is a favored technique for creating CP fibers, such as polyaniline (PANI) emeraldine base fibers, which are utilized in developing artificial muscle fibers due to their robust mechanical and electrical properties (Fig. 6b) [63]. Initially, these PANI fibers exhibit a modest tensile stroke of ∼0.28%. However, when twisted into multifilament yarns, their linear displacement improves to 0.8%, highlighting potential advancements in fiber or yarn-based actuators. To further enhance their functionality, PANI has been doped in HClO_4_ and incorporated into a solid polymer electrolyte matrix to create solid tensile CP-based actuators. Although the tensile stroke remains limited to ∼0.3%, the response time of these actuators can be significantly decreased by reducing the diameter of the devices and using high mobility ions [64].

Another approach has been to coat PANI on inactive fibers or yarns, transforming them into functional artificial muscle fibers by leveraging PANI's superior electrochemical properties as an active functional layer (Fig. 6c). For instance, a unique linear artificial muscle has been developed by coating CP on a hydrogel microfiber. Although the actuation strain in an aqueous electrolyte is relatively modest at ∼0.54%, this muscle is capable of sensing applied current, electrolyte concentration and temperature during its operation [65], highlighting its potential for multifunctional applications in CP-based muscle fibers. In further developments, polypyrrole (PPy) micro-rod actuators have been produced using template synthesis in polycarbonate membranes. Utilizing a specialized microscopy technique coupled with a 3D analysis program, the actuation behavior of these micro-rods can be observed *in situ*, showing they can expand or contract by 20%–30% of their original length when an electrical potential is applied [66]. This miniaturized approach results in a larger actuation stroke compared to larger-scale fibers, likely due to the finer microstructures, which improve ion contact and migration within the CPs. Leveraging miniaturized design for enhanced ion transport, innovative electrochemical actuators have been developed using soft hydrogel nanofibers as templates. These actuators integrate PANI nanostructures onto polyvinyl alcohol (PVA) nanofibers, creating a hybrid mat with a high conductivity of 2.35 S cm^−1^ and a maximum linear stroke of 1.8%. This set-up allows for easy formation of multilayered cylindrical structures, enhancing the surface area and actuation performance [67]. Similarly, hybrid hydrogel nanofibers produced through reactive electrospinning, which combines electrospinning with *in-situ* photopolymerization of hydrogels and aniline polymerization, can finely control fiber properties by adjusting precursor solution concentrations. Lower PANI concentrations lead to faster polymerization, fewer defects and higher doping efficiency, allowing these fibers to quickly respond to low voltage and current, achieving reversible displacements up to 2.5 mm s^−1^ in aqueous electrolyte [68]. This efficient fabrication method shows great potential for applications in wearable e-textiles and other areas requiring rapid actuation.

Recent studies indicated that coil structures have been shown to significantly enhance the performance of linear tensile muscle fibers. By modifying the length and number of PPy-based coil composites, both the tensile stroke and force generation can be tailored to specific needs [69]. For example, reducing the diameter of the coils means a larger proportion of PPy in the cross-section of the fiber composite, which increases the electrochemically generated apparent stress. Additionally, textile techniques such as weaving and knitting improve the functionality of tensile muscle fibers or yarns. For instance, muscles made from cellulose yarns, assembled into fabrics and coated with CPs, exhibit increased force generation and tensile stroke [70]. These developments represent significant strides in the field of electrochemical actuators.

Very recently, innovative pure CP-based artificial muscle fibers have been developed with a unique coil structure, addressing the limitations of conventional CP fiber actuators that often underperform due to the inertness of core or auxiliary materials [71]. Utilizing continuous wet spinning and multiple twisting steps, these high-strength CP microfibers experience significant radial volume expansion due to molecular structural changes during electrochemical processes. This expansion is further amplified by the yarns’ coiled structures, leading to a notable shrinkage strain exceeding 11% under a stress of 5 MPa, which enables the lifting of loads over 4000 times their weight at a low voltage of 1 V.

Collectively, to enhance the performance of CP muscle fibers, several strategic approaches are employed, focusing on optimizing their structural and functional properties. Techniques such as manipulating the physical configuration of the fibers, including spinning them into multifilament yarns and reducing actuator dimensions, significantly improve their actuation stroke and responsiveness. Additionally, innovative coating methods enable these fibers to operate under various environmental conditions and respond to multiple stimuli. Incorporating coil structures further enhances their mechanical performance, allowing for tailored force generation and stoke capabilities. Moreover, integrating textile engineering techniques like weaving and knitting strengthens the mechanical properties and functionality of these fibers.

#### Double-layer capacitance in carbon nano materials

**Figure 7.**
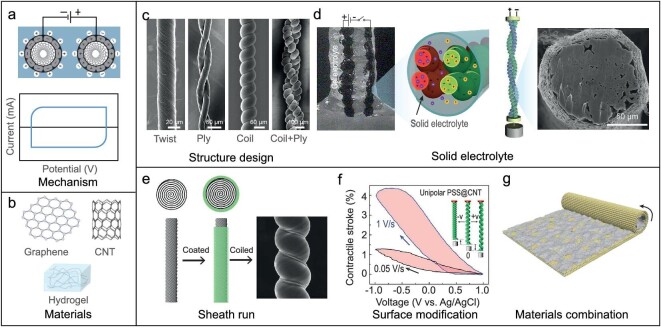
Response mechanisms of artificial muscles based on double-layer capacitance, the representative materials, and their developmental history, including structural design, development of solid electrolytes, *in-situ* material modification and composite formulations. (a) Response mechanisms. (b) Representative materials. (c) Structure design. Reproduced from ref. [123] with permission. Copyright 2014 American Chemical Society. (d) Solid electrolytes. Reproduced from ref. [76] with permission. Copyright 2017 Wiley-VCH. Reproduced from ref. [79] with permission. Copyright 2021 Wiley-VCH. (e) Sheath-run structure. Reproduced from ref. [51] with permission. Copyright 2019 AAAS. (f) *In-situ* material modification. Reproduced from ref. [80] with permission. Copyright 2021 AAAS. (g) Composite formulations. Reproduced from ref. [83] with permission. Copyright 2018 Wiley-VCH.

*Operational principle.* Carbon nano materials, such as CNTs and graphene, utilize the principle of electrical double-layer capacitance for actuation. When an electric field is applied, ions from the electrolyte densely accumulate at the interface of the carbon material, forming an electrical double layer (Fig. 7a). This formation generates electrostatic pressure, mechanically deforming the carbon structure. The efficiency of this process is significantly boosted by the high surface area and superior electrical conductivity inherent to carbon nano materials.

*Material and development progress.* The initial development of electrochemically driven freestanding actuators based on SWCNTs showed promising results with strains exceeding 0.2%, which surpass those of previous CP linear actuators (Fig. 7b) [72]. This led to further innovations, including the development of various oriented CNT-based electrochemical actuators, such as single- and multi-walled CNT pads and aligned ribbons [73]. The discovery of spinnable CNT forests marked the advent of a new generation of CNT-based muscle fibers [74].

CNT yarns drawn from the spinnable CNT forest are initially twisted to introduce a bias angle, which leads to the formation of a regular helix structure, significantly enhancing the actuation performance of these artificial muscle fibers. This structural design enables the yarns to perform both torsional and tensile actuation within a three-electrode electrochemical system [75]. Remarkably, these yarns have demonstrated the ability to undergo reversible rotations of up to 15 000° and achieve ∼1% tensile stroke driving by double-layer charging. To further enhance tensile actuation, additional twisting is applied to create a coiled structure in electrochemically driven CNT yarn muscles. This design enhancement has led to remarkable performance improvements, with these muscles demonstrating tensile contractions of up to 16.5% and a contractile energy conversion efficiency of 5.4% (Fig. 7c) [76]. Based upon these foundational structures, further advancements have been achieved with the development of hierarchically multiply coiled CNT yarn muscles. These muscles exhibit exceptional work capacity and tensile stroke, outperforming mammalian skeletal muscles and functioning as mechanical energy harvesters without the need for external power [77]. Similarly, leveraging these sophisticated structures, electrochemical yarn muscles have been engineered to achieve ultra-large and rapid contractile actions, capable of contracting by 62.4% in just 5 seconds [78].

Because the use of liquid systems or volatile gel electrolytes often limits the practical applications and long-term stability of these muscles (Fig. 7d), all-solid-state muscles is another important research direction in this field. For instance, a gel electrolyte-based muscle has been developed in parallel and braided configurations, which achieve tensile contractions of 11.6% and 5%, respectively [76]. Utilizing another strategy, a pioneering approach involves coating CNT yarn muscles with ionic liquid (IL) encapsulated in nanofibers [79]. These nanofibers act as separators to prevent short-circuiting while also serving as a solid reservoir for the IL. Due to the negligible vapor pressure of IL, the CNT-based muscles can operate reversibly under various challenging conditions, including being knotted, across a wide humidity range (30 to 90 RH%), and at temperatures from 25 to 70°C, even during prolonged cycling and storage in air. This enhanced robustness makes these yarn muscles especially promising for applications in robotic devices.

However, designs allowing for electrolyte penetration throughout the CNT yarn muscle face challenges related to the inefficient use of guest expansion energy and limited mechanical power due to transfer times. To address these challenges, a novel structure known as ‘sheath-run artificial muscles’ (SRAMs) was introduced [51]. In this design, a guest material coats the yarn as a sheath rather than infiltrating it, which significantly enhances the performance in absorption-driven, thermal/electrothermal and electrochemical applications. Specifically, an electrochemically driven CNTs@nylon6 SRAM involves a cylindrical assembly of CNT sheets with nylon yarns centrally positioned. Initially, a twist is applied to the CNT cylinder, which subsequently collapses, forming a tight sheath around the nylon yarn (Fig. 7e). This configuration allows torque to be efficiently transferred from the sheath to the yarn, facilitating full coiling of the yarn. This design significantly enhances performance, with the electrochemical SRAMs achieving 5.2-, 9.0- and 9.0-fold improvements in stroke, contractile work-per-cycle density, and average contractile power density at 1 Hz, respectively, compared to traditional hybrid yarn artificial muscles.

In addition to topological challenges, CNT-based electrochemical artificial muscles face other fundamental limitations, such as a decrease in capacitance when charged rapidly, leading to reduced actuation strokes. Furthermore, these muscles exhibit bipolar behavior during potential scans from extremely negative to positive values, expanding and then contracting, which diminishes the overall stroke. To address these issues, our recent innovations have introduced a unipolar-driven strategy and an electroosmotic pump mechanism (Fig. 7f) [80]. The electroosmotic pumping effect, whereby a large number of solvent molecules are brought into the double-layer region of CNTs when ions migrate to the CNT surface, contributes to the enhancement of the actuation stroke of the CNT muscle. To achieve unipolar ion interaction, we have introduced various materials, including ion polymers, graphene oxide and surfactants, onto the surface of individual CNTs. Unlike bipolar electrochemical actuation, this design enabled unipolar muscles to exhibit a monotonic increase in tensile actuation throughout the entire electrochemical window, with a more than 4-fold increase in stroke. In addition, since increasing the potential scan rate amplified the electroosmotic pumping effect, the enhanced charge transfer rates draw more solvent into the muscle yarn, providing an additional tensile stroke, known as scan-rate-enhanced stroke (SRES). These insights offer new mechanisms and methods for developing more efficient CNT-based electrochemical actuator muscles.

In order to mimic the characteristics of natural muscles, new artificial muscle yarns are expected to have both energy integration systems, yarn structures and tensile actuation capabilities. A multi-walled carbon nanotube (MWNT) coiled yarn was used as a trifunctional yarn, acting as a generator, supercapacitor and actuator simultaneously [16]. This tensile self-powered artificial muscle yarn (SPAM) system incorporates a generator with MWNT coiled yarn (anode) and reduced graphene oxide (cathode). In addition, the MWNT coiled yarn follows the electrochemical actuation mechanism. The coiled yarn structure of SPAM augments the tensile stroke, and the self-charging system enables reversible actuation and rapid contraction within 2.3 s.

Graphene-based electrochemical actuators primarily function through the electrostatic double-layer effect, which stimulates the extension of C-C bonds in carbon rings of graphene through charge injection. Typically, graphene films and fibers produced via vacuum filtration and wet spinning possess a high Young's modulus but lack sufficient toughness, posing challenges in forming highly twisted and coiled structures akin to those of CNT yarn muscles [81]. To overcome this, a novel method involving rod coating and drying of water/graphene oxide (GO) dispersions has been developed to create large-area GO films with enhanced mechanical properties, including significant elongation to fracture and toughness. Following heat treatment and Joule heating, these GO fibers achieve a conductivity of 416 S cm^−1^ and are capable of self-coiling when twisted under a constant load, positioning them as potential high-performance torsional and tensile artificial muscle fibers [82]. Additionally, a new technique using biscrolling has been used to create a CNT/reduced graphene oxide (rGO) hybrid artificial muscle. This process entails depositing rGO nanosheets onto oriented CNT sheets to form a bilayer structure, which is subsequently rolled into yarn and intensely twisted. These coiled CNT/rGO yarn muscles achieve a contraction of 8.1% and a stress of 14 MPa when electrochemically powered, markedly exceeding the performance of natural muscles (Fig. 7g) [83].

**Figure 8.**
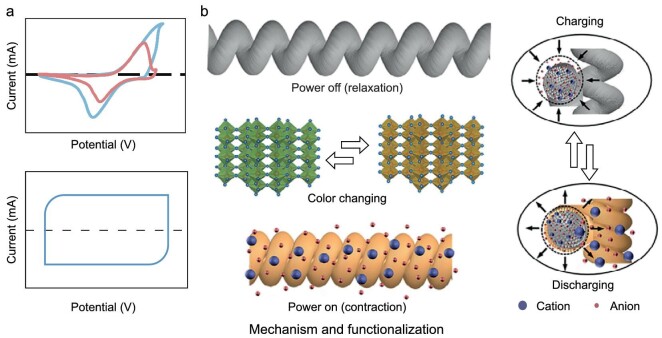
(a) Combining pseudocapacitance and double-layer capacitance for multifunctional artificial muscle fibers. (b) Schematic demonstrating the color change and actuation dual-responsive mechanism of coiled V_2_O_5_ NWs/CNT composite fibers. Reproduced from ref. [88] with permission. Copyright 2023 Wiley-VCH.

*Combining pseudocapacitance and electric double-layer capacitance.* The integration of pseudocapacitive materials with electric double-layer materials (Fig. 8a) has proven to be a highly effective approach for enhancing the actuation and mechanical performance of composite muscle fibers. This synergy addresses the common limitations of double-layer capacitive electrochemical actuators, such as small driving strokes and low output forces [84].

A recent study involved the preparation of PPy/graphene fiber-based electrochemical muscle fibers through the electropolymerization of pyrrole on graphene fibers. This muscle not only exhibited high actuation activity and durability but also demonstrated potential applications in biological research and micromachining [85]. Additionally, CNT@PANI composite coiled yarns with a core-shell structure were developed by incorporating PANI into CNT narrow ribbons through soaking and *in-situ* polymerization [86]. The CNT provided the yarns with excellent flexibility, while the reversible redox reactions of pseudocapacitive PANI increased capacitance and induced further deformation. The synergistic effects of double-layer capacitance of CNT and pseudocapacitance of PANI, combined with the coiled geometry, allowed these composite muscles to achieve a contraction stroke of 17%—approaching the 20% contraction stroke of human skeletal muscle—and contraction stress of 8 MPa, which is significantly higher than the 0.35 MPa typical of skeletal muscle, at low driving voltages (<2 V) and in 0.01 M phosphate buffered solution simulated body fluid environments. Remarkably, these artificial muscles maintained exceptional properties in biocompatible solutions like normal saline and Na_2_SO_4_ aqueous solution, underscoring their suitability for implantable bionic medicine applications.

Moreover, the development of multifunctional electrochemical artificial muscles through this combination strategy has led to the creation of innovative electrochromic coil yarns. These yarns combine CNT yarns with V_2_O_5_ as a core material and a functional guest sheath, fabricated via a unique fluidic spinning process [87]. Such metal oxides are based on the intercalation pseudocapacitance mechanism, where lattice contraction/recovery during the mobile cations de-/intercalation is accompanied by color changes (Fig. 8b), for achieving dual-response capabilities. These dual-responsive electrochemical muscles demonstrated a 15.3% tensile stroke and a working capacity of 0.82 J g^−1^ through electrochemical charge injection, showcasing their utility in electrochromic muscle. Further enhancing the functionality, a similar strategy was used to develop another type of electrochromic artificial muscle. This muscle incorporates a core-sheath structure combining V_2_O_5_ nanowires with CNT fibers, tightly bonded by wet twisting, which acts as the inner actuation source. This dual-responsive electrochromic artificial muscle (EAM) has proven to operate stably in air, producing a contraction stroke of 12% and displaying multiple color changes (yellow-green-gray) at a voltage of ±4 V (Fig. 8b) [88]. Additionally, a fast-responding toroidal EAM arrangement was constructed, capable of displaying visual color changes that represent contraction strokes. These advancements underscore the significant potential of merging pseudocapacitance with electric double-layer capacitance to develop next-generation electrochemical muscle fibers.

**Figure 9.**
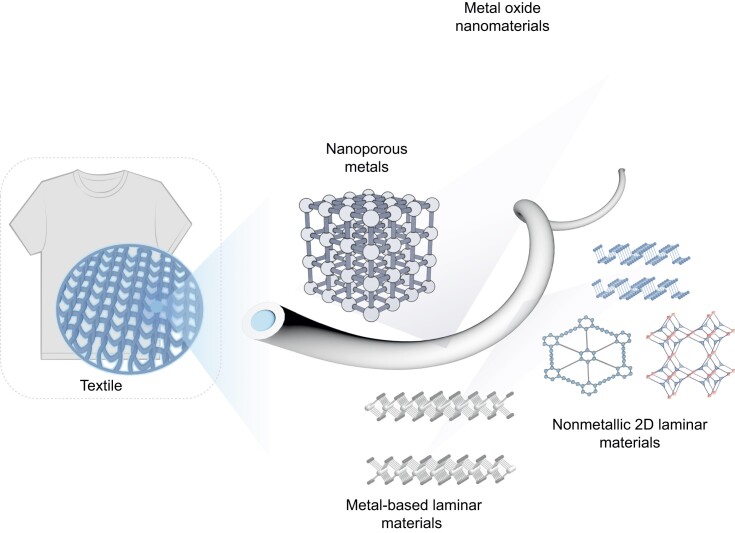
Materials and actuation mechanisms of electrochemical muscle fibers.

*Exploring material diversity and actuation mechanisms in electrochemical muscle fibers.* In the realm of electrochemical muscles, the diversity of nanometal-based materials contributes to the complexity of their actuation mechanisms (Fig. 9). Notably, nanoporous noble metals exhibit significant volume contraction and expansion due to charge-transfer-induced surface stress changes, enhanced by the high surface area of the nanoporous structure [89,90]. Additionally, redox pseudocapacitive processes are prevalent in non-precious metals or metal oxide materials such as MnO_2_ and RuO_2_ [91,92], while ion intercalation pseudocapacitance involves the insertion of alkali metal ions or protons between the atomic layers of laminar metal-based materials, primarily transition metal dichalcogenides. This process results in a reversible expansion of the material's lattice or interlayer spacing, leading to mechanical actuation [93,94].

Furthermore, novel electrochemical actuators utilizing non-metallic 2D laminar materials like covalent organic frameworks, graphdiyne and black phosphorous also exhibit similar actuation mechanisms [95–97]. These materials, along with those previously mentioned, can be integrated into ionic polymer-metal composites, which consist of an ion-exchanged membrane sandwiched between two electrically conducting electrodes [98,99]. However, despite the remarkable advancements and innovative designs in electrochemical actuators, the application of these materials in fiber configurations remains underdeveloped. This is primarily due to the inherent high Young's modulus and strength of the materials, which pose challenges in fiber development, as well as the limitations related to production techniques and costs.

### Dielectric actuation

**Figure 10.**
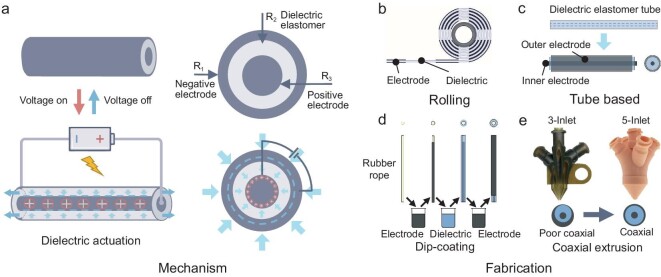
Working principles of dielectric elastomer artificial muscle fibers and their fiber formation methods. (a) Working principles. (b) Rolling process. Reproduced from ref. [106] with permission. Copyright 2004 IOP Publishing Ltd. (c) Tube structure. Reproduced from ref. [112] with permission. Copyright 2006 Elsevier B.V. (d) Multilayered tube structure. Reproduced from ref. [114] with permission. Copyright 2010 Springer-Verlag. (e) Multicoaxial 3D printing process. Reproduced from ref. [118] with permission. Copyright 2021 Wiley-VCH. Reproduced from ref. [119] with permission. Copyright 2023 American Chemical Society.

Dielectric (DE) actuation, leveraging the unique properties of DE elastomers, represents a significant area of research and application within the field of electrically powered artificial muscles. This technology utilizes electric-field-induced deformation to achieve actuation, offering a compelling combination of efficiency [100], scalability and versatility [100–102]. Dielectric elastomer artificial muscle (DEAM) actuation operates on the principle of electric-field-induced deformation in DE elastomers. When a voltage is applied across a DE elastomer, a large amount of positive and negative charges accumulate on the positive and negative electrodes. Due to electrostatic forces, the positive and negative charges attract each other, resulting in Maxwell stress, causing the DE elastomer to compress and be thinner along the direction of the electric field, and expand perpendicular to the direction of the electric field (Fig. 10a) [103,104]. This deformation process is reversible, allowing the material to return to its original shape once the electric field is removed. The actuation process directly converts electric field energy into mechanical energy, resulting in relatively high energy efficiency. The key to this mechanism is the DE material's ability to undergo significant strain while maintaining high levels of energy density and efficiency. However, common elastic materials such as polyacrylates and silicone have high viscoelastic losses, making it difficult to further improve the actuation efficiency. An innovative solution is to introduce hydrogen bonds into the polymer network [105], which not only reduces viscoelastic losses, but also adjusts the stress-strain response of the material and improves stability under large deformations. This further enables DE materials to possess high energy density, efficiency and large deformation characteristics simultaneously.

A significant study demonstrated that materials like acrylic and silicon elastomers could achieve over 100% strain and extremely high energy density under electric field stress, highlighting the unique advantages of the DE actuation mechanism [104]. This spurred a wave of research, primarily centered on film structures. However, film-based DEAMs, while easily manufactured at high throughput, struggle with forming complex 3D structures or miniaturization. Furthermore, the biaxial area strain typically produced by thin film DEAM often requires additional structural design for practical application. Thus, DE elastomer artificial muscle fibers (DEAMFs), which offer linear strain similar to natural muscle fibers, have shown their distinct advantages.

The initial transformation of DEAM muscle from film to fiber involved mechanically simple but technically challenging methods. Initially, films were rolled into fibers, creating spiral structured DEAMFs (Fig. 10b). An early method wrapped pre-stretched acrylic films around cylindrical springs to produce linear strain DEAM, forming a fiber-like structure [106,107]. However, this approach required springs to maintain axial tension, complicating the fabrication process and limiting the production of finer fibers. Advances in this area have included the spin-coating of silicone elastomer films layered with CNT electrodes before being wound into fibers, proving that the pre-stretching process is not essential [108]. The DEAMFs manufactured by this method are used to drive micro flying robots and pipeline robots [109,110]. Despite these advancements, rolling this sub-millimeter-thick film into small diameter fibers remains a challenge, and this hollow structure has unnecessary radial strain. To address these challenges, a thermomechanical treatment was developed to adjust the film's orthogonal modulus ratio, enabling the creation of micrometer-level anisotropic thin films. These films could then be tightly wound into millimeter-scale fibers [111], significantly reducing radial strain and enhancing axial strain, although the technique was still limited to producing relatively short fibers.

For longer DEAMFs, a coaxial structure using commercial elastic tubes was introduced, with electrodes and DEs arranged in alternating layers. This design utilized polyurethane and silicone tubes, filled with a calcium chloride solution as the inner electrode and coated externally with carbon-black-doped silicone (Fig. 10c) [112], achieving ∼7% axial strain. This set-up proved simple but limited in customizing DE materials. Innovative solutions for material customization included forming DE tubes through injection molding of the mixture of polydimethylsiloxane (PDMS) and silicone elastomer for applications such as underwater robotics [113], or sequentially dip-coating electrodes and DEs onto a rubber rope (Fig. 10d) [114], which was later removed to create the DEAMFs. A more continuous production method used coaxial wet spinning to produce PDMS hollow long fibers in an ethanol bath, employing IL and gels as internal and external electrodes [115,116]. To further streamline and automate DEAMFs production, a double-layer extrusion head was developed for melt coextrusion. This process involved coextruding thermoplastic polyurethanes and graphite-doped PDMS [117], with graphite subsequently applied manually to the outer layer to form the DEAMFs. The above methods can already produce DEAMF in a continuous and integrated manner, but processing DEAMFs into complex structures still poses challenges. A novel approach, combining coextrusion with DIW 3D printing, employed a multicore-shell 3D printing method for continuous and integrated fabrication of DE elastomer fibers, greatly expanding the application forms of DEAMFs [118]. This method utilized a three-layer coaxial printhead to coextrude three different material layers, where the printing material was based on silicone and PDMS, and the electrode material was doped with carbon black. This DEAMF could form multiple strands of fibers or coils through 3D printing, showcasing a resonance frequency up to 700 Hz and a lifespan of up to 2.6 million cycles. However, the fibers produced by this method have defects in coaxiality. One method is to increase and symmetrically arrange the feeding ports of the printing head, which improved the axiality between the layers of extruded fibers and led to a 35% increase in the DE breakdown strength of DEAMFs (Fig. 10e) [119]. This advanced printing method was utilized to fabricate a coil-shaped DEAMF, which was incorporated into a high-speed, insect-scale robot achieving movement frequencies up to 760 Hz. Notably, doping the surface-modified zinc oxide nanoparticles into multi-block copolymers can significantly improve the DE breakdown strength [120], and surface-modified BaTiO_3_ can greatly enhance the electromechanical conversion performance of polyurethane [121]. These have been demonstrated on thin film DEAM, pointing towards potential applications in DEAMFs as well.

## CONCLUSION AND PERSPECTIVES

In summary, the field of EAMFs has witnessed transformative advancements, propelled by the meticulous exploration of electrothermal, electrochemical and DE actuation mechanisms. Each mechanism has carved out new pathways for the development of highly responsive, efficient and biomimetic artificial muscles. Table 1 reveals significant performance variations among artificial muscle fibers based on different actuation mechanisms and materials. For instance, electrothermal artificial muscle types incorporating high-strength fibers such as nylon and SMA can typically output greater mechanical energy. However, their strong dependency on thermal management restricts their actuation frequency range. In contrast, electrochemical actuation mechanisms, relying on ion storage response mechanisms, exhibit higher efficiency compared to other types of fibers. Yet, their dependency on liquid or gel-state electrolytes limits their adaptability to different operating environments. Dielectric elastomers, utilizing electrostatic attraction mechanisms, can enhance their operating frequency quickly through the rapid application of voltage, offering broad application prospects in domains requiring high-frequency motion.

**Table 1.** Mechanism, performance, advantages, limitations and applications of current categories of artificial muscle fibers.

Mechanisms	Typical materials	Performance (optimum values)		Ref.	Advantage	Limitation	Application
Electrothermal actuation	Ordinary polymers Shape memory materials LCE Carbon-based nanomaterials	Stroke Stress Work capacity Bandwidth Cyclability	∼60% >100 MPa >2.7 kJ kg^−1^ <25 Hz 10^6^	[58] [55] [25] [15] [58]	Low cost High power density High response rate High cycling stability	Low efficiency Low security Thermal management challenges	Wearable technology [37,40,41,59] Biomedical devices [58] Soft robotics [35,36,39,124]
Electrochemical actuation	Pseudo-capacitive materials (CPs)	Stroke Stress Work capacity Bandwidth Cyclability	∼30% >10 MPa 0.3 kJ kg^−1^ <1 Hz 2.5 × 10^3^	[66] [71] [71] [71] [70]	Wide stroke range Good biocompatibility	Low strength Low stiffness Low cyclic stability High cost	Wearable electronics [70,125] Biomedical devices [126] Energy storage [127] Soft robotics [71]
	Electric double-layer capacitor (EDLC) (carbon-based nanomaterials)	Stroke Stress Work capacity Bandwidth Cyclability	>60% 60 MPa 4.1 kJ kg^−1^ <10 Hz 10^4^	[78] [51] [80] [80] [51]	Lightweight, high response rate High work capacity Good cycling stability	High cost	Soft robotics [79] Biomedical and bionic devices [79,128]
Dielectric actuation	DE	Stroke Stress Work capacity Bandwidth Cyclability	∼12% ∼15 N 4.32 J kg^−1^ >700 Hz 2.6 × 10^6^	[118] [106] [111] [119] [118]	High response rate High efficiency Wide response range Low power consumption	High driving voltage (kV level)	Soft robotics [113,119]

Given the strengths, limitations and application potential of each mechanism in artificial muscle technology, it is possible to outline several prospects for future developments.

### Vision for electrothermal artificial muscle fibers

Owing to the electrothermal response mechanism, the effective control of heat absorption and dissipation is a primary factor for the precise actuation of electrothermal artificial muscle fibers. However, there is currently a paucity of work on thermal management for electrothermal artificial muscle fibers. This limitation could severely restrict the future practical application of electrothermal artificial muscle fibers.

Innovations in thermal management for optimal efficiency: initially, by refining the structural design, the interface between electrothermal heating elements and thermally responsive materials can be improved, enhancing the fiber's rapid response to thermal stimuli. Integrating phase change materials into the fiber structure enhances thermal control by modulating heat absorption and release during phase transitions, stabilizing the fiber's temperature during operation, and boosting energy efficiency. Furthermore, deploying portable fluid circulation systems provides an innovative solution for managing heat distribution, maintaining even temperatures across the fibers, preventing overheating and facilitating rapid thermal adjustments. These strategies may drive the development of more capable and responsive electrothermal artificial muscle fibers, improving their efficiency, durability and adaptability to a variety of cutting-edge applications.

### Vision for electrochemical artificial muscle fibers

Fiberization of high capacitance nanomaterials: the integration of novel, low-dimensional nanomaterials with high capacitance properties is transforming electrochemical artificial muscle fibers. These materials, pivotal in energy storage applications, present significant opportunities for enhancing muscle fiber functionality. The challenge lies in converting these materials into fibrous forms without diminishing their structural integrity or electrochemical efficacy. Mastering this process will significantly enhance the mechanical strength and functional performance of electrochemical artificial muscles, enabling more sophisticated applications.

Advancements in solid-state electrolytes and new mechanisms: developing solid-state electrolytes represents a critical focus area, moving beyond semi-solid artificial muscles using gel electrolytes that depend on solvents or other liquids. Advancing towards truly solid-state electrochemical artificial muscle fibers offers substantial benefits, including enhanced mechanical properties and stability. Additionally, enhancing the volumetric change induced by unit ion injection remains a priority. Increasing capacitance to facilitate greater ion injection can lead to enhanced volumetric changes, but overcoming the inherent capacitance limitations of capacitive materials is a critical challenge. Future research should investigate methods to increase the volumetric occupancy of individual ions, which is expected to substantially improve the performance of artificial muscles by deepening our understanding of ion dynamics within artificial muscle systems.

Functional integration and design innovations of electrochemical artificial muscle fibers: integrating electrochemical artificial muscles with electrochemical energy storage is a primary focus for future research, aiming to enable artificial muscle fibers to function both as mechanical energy output components and as electrical energy storage devices. From a design perspective, textile techniques such as weaving and knitting are crucial for structuring electrochemical muscle fibers. A key challenge is developing a weaving structure that integrates working and counter electrodes into a single piece of fabric without mutual interference, facilitating seamless and efficient function.

### Vision for dielectric elastomer artificial muscle fibers

The development of DEAMFs has been significantly influenced by advancements in manufacturing technologies. In particular, 3D printing stands out as a pivotal manufacturing technique that significantly enhances the design and continuous production capabilities for these types of artificial muscles. However, the current robots based on DEAMFs all require connection to a power source due to their high voltage demands, which greatly limits their development in the field of miniaturized robots. Therefore, developing novel manufacturing processes for creating DEAMFs suitable for portable power sources is crucial for expanding their application scope in the future.

### Vision for the application of electrically powered artificial muscle fibers

Electrically powered artificial muscle fibers have been applied across multiple domains, with notable preliminary explorations particularly in smart wearables and soft robotics [40,50,122]. These applications illustrate the versatility and potential of these fibers in enhancing the functionality and adaptiveness of various technologies. For example, innovations like SMA-based soft grippers, which adjust stiffness using paraffin wax, enable highly precise and powerful gripping actions [35]. Similarly, using shape memory alloy wires with constrained braiding techniques enhances actuation in substrate-free robots, making them ideal for precise bending in 3D-printed and origami robots to improve locomotion [42]. In aquatic environments, a jellyfish-like robot built with a DE elastomer muscle fiber—a silicone tube filled with a sodium chloride solution—achieved a swimming speed of 3.1 mm/s at a 4 Hz driving frequency [120]. Additionally, recent research in ultrafast, insect-scale soft robots using coil DE elastomer muscle produced through multi-material coaxial 3D printing has demonstrated high robustness and adaptability, excelling in navigating complex terrains and participating in swarm tasks [113].

Overall, while artificial muscle fibers boast considerable advantages over traditional actuators, there are still areas requiring improvement, including precision control, mechanical output and operational frequency range. Recent studies have focused on addressing these challenges, resulting in significant advancements. Looking ahead, it is essential to thoroughly investigate the specific actuation characteristics of these fibers and broaden their application scope to maximize their unique properties. Furthermore, by advancing the integration of actuation and sensing within these fibers and leveraging cutting-edge textile technologies such as programmable jacquard weaving, we can enhance the programmability of artificial muscle fibers at the textile level. This development significantly boosts their applicability in wearable technologies, expanding the possibilities within smart textiles-based devices.
